# Type III, IV, and VI Collagens Turnover in Systemic Sclerosis – a Longitudinal Study

**DOI:** 10.1038/s41598-020-64233-8

**Published:** 2020-04-28

**Authors:** Pernille Juhl, Anne-Christine Bay-Jensen, Roger Hesselstrand, Anne S. Siebuhr, Dirk M. Wuttge

**Affiliations:** 1 Department of Biomedical science, Copenhagen University, Copenhagen, Denmark; 2 Biomarkers and Research, Nordic Bioscience, Herlev, Denmark; 3 Department of Clinical Sciences Lund, Rheumatology, Lund University and Skåne University Hospital, Lund, Sweden

**Keywords:** Biomarkers, Skin diseases

## Abstract

Tissue turnover, especially in the skin, is altered in systemic sclerosis (SSc), leading to tissue accumulation. The objective was to examine type III, IV, and VI collagens turnovers in SSc and investigate longitudinal alterations in relation to modified Rodnan Skin Score (mRSS). We included patients fulfilling the 2013 ACR/EULAR criteria for SSc (limited cutaneous [lcSSc, n = 20], diffuse cutaneous SSc [dcSSc, n = 23]) and healthy controls (HC, n = 10). Biomarkers of type III, IV, and VI collagens formation (PRO-C3, PRO-C4, PRO-C6) and degradation (C3M, C4M, C6M) were measured in serum. The fibrotic index of the individual collagens (FICol) were calculated. The fibrotic index of type III and VI collagens (FICol3 and FICol6) were increased in dcSSc compared to lcSSc (FICol3: 1.4 vs. 0.8, P = 0.0001; FICol6: 1.2 vs. 0.9, P = 0.03). The fibrotic index of type IV collagen (FICol4) was not different between the groups but was 1.5 times higher than HC (HC: 6.9, lcSSc 10.4, dcSSc: 10.5). Both FICol3 and FICol6 correlated with mRSS with rho’s of 0.59 (P < 0.0001) and 0.35 (P = 0.04). Furthermore, FICol3 steadily decrease over the disease course. Examining collagen turnover and specific collagens could be beneficial in following patients’ skin fibrosis and possibly identifying progressors.

## Introduction

Systemic sclerosis (SSc) is a dynamic disease in which the literature suggests that microvascular damage leads to damage to endothelial cells resulting in fibrosis by activation of fibroblasts^1,2^. Fibrosis is both seen around the blood vessels and in the deeper interstitial matrix of the affected tissues. The majority of SSc patients have skin fibrosis, whereas some have fibrosis of various internal organs. The extent of skin fibrosis is used to divide patients into two subgroups; limited cutaneous SSc (lcSSc) and diffuse cutaneous SSc (dcSSc)^3^.

Fibrosis is the result of a tilted remodelling of the extracellular matrix (ECM). Fibroblasts, together with other cells, are responsible for maintaining the ECM by ensuring that damaged proteins, such as collagens, are degraded and replaced by new collagens. If fibroblasts are continuously activated, they will differentiate into myofibroblasts and produce more ECM proteins than needed resulting in fibrosis.

Collagens are the most abundant protein group in the body, where different collagens have different roles and locations. The synthesis of collagen is known to be upregulated in SSc, and the focus of the literature has primarily been on the two main collagens found in skin; type I and III collagens. These are usually found in the interstitial matrix, where they act as the “skeleton” of tissue as the skin. Other collagens as type VI collagen is furthermore found in the interstitial matrix of the skin where it is implicated in matrix organization and fibrillogenesis of collagens^4^. Type VI collagen gene and protein levels have been shown upregulated in SSc^5,6^. The ECM composition of the basement membrane around blood vessels differs from the interstitial matrix. The basement membrane mainly consists of type IV collagen and laminin compared to the type I and III collagens-rich interstitial membrane. Type VI collagen is primarily found in the intersection between the basement membrane and interstitial matrix and throughout both sections. Knowledge of different collagens can help understand the location of the fibrosis and target specific types of fibrosis.

The study aimed to examine collagen turnover in SSc patients by investigating the level of formation and degradation of ECM metabolites in blood. Furthermore, we studied the change in collagen turnover over time and in relation to the modified Rodnan Skin Score (mRSS).

## Patients and Methods

### Study population

In this cross-sectional and longitudinal pilot study, we included 43 SSc patients fulfilling the 2013 ACR/EULAR criteria^7^. Twenty patients had lcSSc and 23 patients fulfilled the criteria for dcSSc^3^. All patients had multiple visits to the department of rheumatology, Skåne university hospital-Lund (3–11 visits) where blood samples and clinical features were obtained^8^. Non-medicated asymptomatic controls (n = 10) were included. Serum samples were collected and stored at −80 °C until measured. The study was approved by the Swedish Ethical Review Authority in Lund (approval number Dnr 590/2008) and carried out following the Declaration of Helsinki. All participants had given written, informed consent.

### Serological protein fingerprint biomarkers for ECM turnover

ECM turnover was examined by assessing biomarkers of type III, IV, and VI collagens formation (PRO-C3^9^, PRO-C4^10^, and PRO-C6^11^, respectively) and degradation (C3M^12^, C4M^13^ and C6M^14^, respectively) (produced by Nordic Bioscience). Validated competitive ELISAs assessed analytes according to the manufacture’s protocols. Measurements below the lower limit of detection were assigned the value of the lower limit of detection.

The turnover (fibrotic index) was calculated to examine collagen deposition. It was calculated by dividing the collagen formation with the degradation biomarker.

$${\rm{Fibrotic}}\,{\rm{index}}\,{\rm{of}}\,{\rm{type}}\,{\rm{x}}\,{\rm{collagen}}=\frac{Type\,x\,formation\,(PRO-Cx)}{Type\,x\,degradation\,CxM)}\,,$$

x indicating the collagen number.

### Statistics

Summary statistics were used to examine the baseline demographics at their first visit. Data are depicted as mean and standard deviation (SD) or numbers and percent (%). As the biomarkers were not normally distributed, non-parametric tests were used. The difference in biomarker levels between lcSSc and dcSSc was tested by the Mann-Whitney U test and correlations were evaluated by spearman’s correlation. Duration and SSc specific differences in the levels of the measurements (mRSS or biomarkers) were analysed using Generalized Additive Mixed Models^15^. We used a quasi-poisson distribution as a model of the probability distribution of mRSS measurements, and a gamma distribution as a model of the probability distribution of biomarker measurements. To account for the repeated measurements within patients we used an exchangeable correlation structure. The expected levels of the measurements were smoothed using thin plate splines with automatic smoothing selection^16^.

For each measurement we consider three nested model

- Base model: Smooth effect of duration, no SSc-form differences
- General model: Separate smooth effects duration age by the two SSc-forms. The base and general models were compared by likelihood-ratio tests to assess the hypothesis of no SSc-form differences in the level and shape of the timecourse of the measurements.

The biomarker levels were displayed as mean. *P*-values ≤ 0.05 were considered statistically significant. Graphical illustrations and Mann-Whitney U test were performed using GraphPad Prism version 8, spearman’s correlation was analysed by MedCalc statistical software version 18.11.6, and the analyses and graphics of Generalized Additive Mixed Models were performed in R version 3.6.1^17^.

## Results

The patients were subdivided based on the extent of skin involvement and the demographic data analysed (Table 1). The lcSSc subgroup had longer disease duration and lower mRSS, as expected. Otherwise, the two subgroups were similar regarding age, BMI, and the patients being predominately females.

**Table 1:** Baseline characteristics of limited and diffuse cutaneous SSc and healthy controls.

	Healthy controls (n = 10)	Limited cutaneous SSc (n = 20)	Diffuse cutaneous SSc (n = 23)
Age, years	50 (9)	54 (14)	53 (16)
Female, %	7 (70)	16 (80)	18 (78)
BMI, kg/m^2^		24.4 (4.7)	24.8 (4.1)
Disease duration, months		32.3 (41.0)	13.5 (14.9)
MRSS		4.3 (3.1)	20.3 (11.7)
**Organ involvement**			
Pulmonary fibrosis		11 (55)	9 (39)
**Treatments**			
Calcium antagonist, %		9 (45)	8 (35)
ACE inhibitor, %		1 (5)	3 (13)
Iloprost, %		0 (0)	1 (4)
Prednisolone, %		3 (15)	6 (26)
Mycophenolate mophetil, %		1 (5)	0 (0)
Azathioprine, %		1 (5)	0 (0)
Methotrexate, %		1 (5)	0 (0)

BMI: Body mass index. mRSS: Modified Rodnan skin score. Data are shown as numbers (percent) or mean (standard deviation).

### Collagen turnover in SSc subgroups

**Figure 1:**
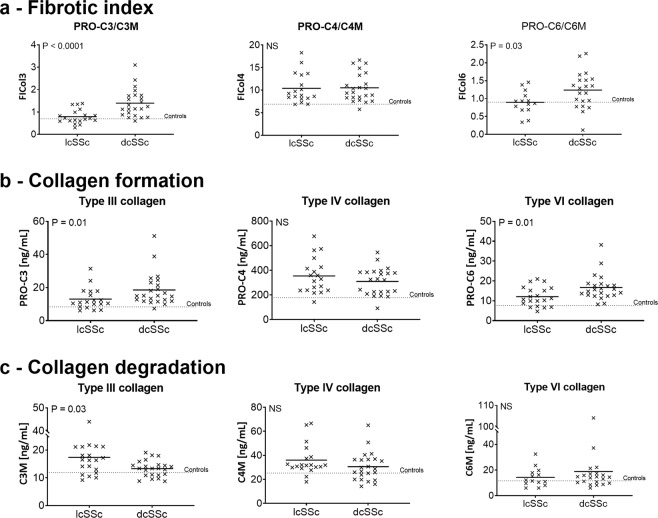
Fibrotic index of type III, IV, and VI collagens in limited and diffuse SSc. (**a**) cross-sectional view of the fibrotic index of type III collagen. (**b**) Cross-sectional view of the fibrotic index of type IV collagen. (**c**) Cross-sectional view of the fibrotic index of type VI collagen. Data are shown as mean. Data were analysed by the Mann-Whitney U test. *P < 0.05, **P < 0.01, ***P < 0.001.

Collagen turnover was examined at the first time point to examine levels in the two subgroups of SSc. The turnover of type III (FICol3) and VI (FICol6) collagens were increased in dcSSc compared to lcSSc (FICol3; lcSSc (mean): 0.8, dcSSc (mean): 1.4, P = 0.0001, FICol6; lcSSc: 0.9, dcSSc: 1.2, P = 0.03; Fig. 1). For both collagens, the level in lcSSc was similar to healthy controls. Type IV collagen turnover (FICol4) was not different between the two SSc subgroups (lcSSc: 10.4, dcSSc: 10.5). However, it was approximately 1.5 times higher than in the healthy controls. The collagen turnover is a product of formation and degradation. Formation and degradation were also examined separately. Type III and VI collagens formation were significantly higher in dcSSc compared to lcSSc (PRO-C3; lcSSc: 31.4 ng/ml, dcSSc: 51.2 ng/ml, P = 0.01, PRO-C6; lcSSc: 4.7 ng/ml, dcSSc: 8.2 ng/ml, P = 0.1). Type III collagen degradation was significantly increased in lcSSc compared to dcSSc (lcSSc: 17.4 ng/ml, dcSSc: 13.3 ng/ml, P = 0.03) and type VI collagen degradation was (lcSSc: 14.3 ng/ml, dcSSc: 18.8 ng/ml) was not different in the two SSc subsets. Type IV collagen formation (lcSSc: 354.4 ng/ml, dcSSc: 310.3 ng/ml,) showed a tendency for a small increase in lcSSc compared to dcSSc and the same was seen in type IV collagen degradation (lcSSc: 36.1 ng/ml, dcSSc: 30.6 ng/ml).

### Type III and VI collagens correlates with mRSS

The levels of formation, degradation and turnover of type III, IV and VI collagens correlated with mRSS at first time point. Type III collagen formation (PRO-C3) and turnover (FICol3) showed the best correlations with Rho’s of 0.53 (P = 0.0003) and 0.59 (P < 0.0001), respectively. Type VI collagen formation and turnover followed with Rho’s of 0.40 (P = 0.008) and 0.35 (P = 0.04), respectively. Type IV collagen formation and turnover did not correlate with mRSS (PRO-C4: Rho = −0.07, FICol4: Rho = 0.17). Collagen degradation biomarkers did not correlate with mRSS (C3M: Rho = −0.16, C4M: Rho = −0.14, C6M: Rho = 0.04).

### Type III collagen changes over time

**Figure 2:**
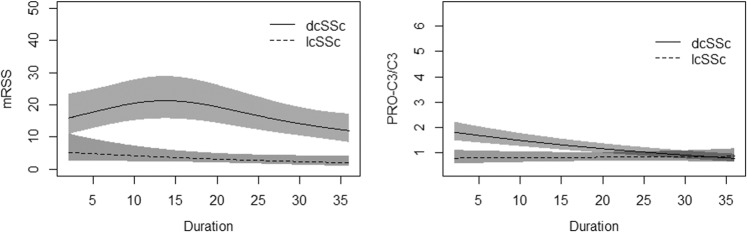
Modified Rodnan skin score and the fibrotic index of type III collagen in lcSSc and dcSSc. Data are shown as mean. Data were analysed by Generalized Additive Mixed Models^15^.

Type III and VI collagens formation and turnover correlated moderately with mRSS at a cross-sectional measure, and we were therefore interested in the changes over time. Serum samples at different time points were collected for each patient. However, patients did not have the same time points. MRSS was higher in dcSSc compared to lcSSc, especially at the beginning of the disease (Fig. 2). It reached a peak between 10 and 20 months where after it decreased. Type III collagen turnover furthermore observed a difference between lcSSc and dcSSc over time. DcSSc had high levels of type III collagen turnover at the beginning, which steadily fell over the disease duration.

## Discussion

In this study, we examined the blood levels of formation, degradation, and turnover of type III, IV, and VI collagens metabolites and their relation to mRSS. We found that collagen turnover was better at separating SSc subsets than single collagen biomarkers and that type III and VI collagens, but not type IV collagen, correlated with mRSS. From these data, we argue that it is crucial to analyse collagen turnover and individual collagen types as type III, IV, and VI collagens.

This study analysed SSc patients according to their skin involvement, i.e., lcSSc and dcSSc. The dcSSc patients are known to have increased fibrosis, not only of the skin but also internal organs. This fits with our data showing increased collagen production in this patient subset. The dcSSc patients had increased type III and VI collagens turnover compared to lcSSc patients. We have previously examined the formation and degradation of collagens and seen that especially collagen formation is upregulated in SSc^6,18^. Increased skin thickness is likely due to increased collagen production. Both type III and VI collagens turnover correlated moderately to strongly with mRSS. The correlation supports the hypothesis that type III and VI collagens is related to skin fibrosis. The formation biomarkers of type III and VI collagens were also elevated in dcSSc patients and correlated moderately with mRSS. However, with increased formation, there might also be increased degradation, i.e., an overall higher turnover. We have previously shown that degradation biomarkers can be elevated in dcSSc patients^6,19^. We, therefore, argue that the collagen turnover gives a more accurate picture of the collagen deposition.

Type III and VI collagens are known to be in the interstitial matrix throughout the dermis^20^. Type IV collagen is found in the basement membrane around blood vessels and in the dermo-epidermal junction^21^. Contrary to type III and VI collagens, type IV collagen did not differ between lcSSc and dcSSc; it was elevated in both subgroups compared to controls. This indicates that type IV collagen turnover is overall elevated in SSc patients but not related to subtypes. Furthermore, type IV collagen turnover did not correlate with mRSS. A hallmark of SSc is vascular damage, especially microangiopathy^22^. Type IV collagen remodelling is affected by constant vascular damage. As vascular damage is found in both lcSSc and dcSSc, it might explain the increase of type IV collagen turnover in both lcSSc and dcSSc compared to controls. Type IV collagen has previously been shown upregulated in SSc by Motegi and colleagues^23^. They found that type IV collagen was elevated in patients with three years of disease duration or less. This corresponds well with our findings as our average disease duration was below three years. We have previously shown that type IV collagen formation and degradation were elevated in dcSSc patients compared to controls, especially in early SSc (<2 years of disease duration)^6^. Overall, these three studies show that there is an increased amount of type IV collagen with SSc and that its potential and meaning should be further examined.

Longitudinal samples showed a trend for type III collagen turnover to change over time in dcSSc. This indicates that type III collagen changes throughout the disease and could offer insight into the pathogenesis. It was increased in the first 20 months of the disease, similar to mRSS. However, instead of having a peak, the levels steadily fell over the disease course. Type III collagen turnover, therefore, shows that serologically measured biomarkers could help understand the changes in disease over time. These data are preliminary and needs to be validated.

The limitations of this study include that different treatments may have affected biomarker levels. Nevertheless, analysis of type III collagen turnover mirrored the widely used clinical readout mRSS throughout the disease.

In conclusion, type III and VI collagens are correlated with mRSS and elevated in dcSSc patients. Type III collagen furthermore changes over time, indicating that especially type III collagen turnover can be used as a biomarker of skin fibrosis. Type IV collagen turnover is not related to mRSS but elevated in SSc patients, suggesting that it might be useful for vascular involvement and not skin fibrosis. Collagen turnover might have additional power compared to only looking at formation or degradation biomarkers. This opens the field for following patients with serological biomarkers.

## Data Availability

The datasets generated during and/or analysed during the current study are available from the corresponding author on reasonable request.
